# Graphic analysis of flow-volume curves: a pilot study

**DOI:** 10.1186/s12890-016-0182-8

**Published:** 2016-01-22

**Authors:** Jungsil Lee, Choon-Taek Lee, Jae Ho Lee, Young-Jae Cho, Jong Sun Park, Yeon-Mok Oh, Sang-Do Lee, Ho Il Yoon, KOLD Study Group

**Affiliations:** Department of Internal Medicine, Seoul National University College of Medicine, Division of Pulmonary and Critical Care Medicine, Seoul National University Bundang Hospital, 166, Gumi-ro, Bundang-gu, 463-707 Seongnam-si, Gyeonggi-do Republic of Korea; Department of Pulmonary and Critical Care Medicine and Clinical Research Center for Chronic Obstructive Airway Diseases, Asan Medical Center, University of Ulsan College of Medicine, Seoul, Republic of Korea

**Keywords:** Pulmonary disease, Chronic obstructive, Maximal expiratory flow-volume curves, Spirometry

## Abstract

**Background:**

Conventional spirometric parameters have shown poor correlation with symptoms and health status of chronic obstructive pulmonary disease (COPD). While it is well-known that the pattern of the expiratory flow-volume curve (EFVC) represents ventilatory dysfunction, little attempts have been made to derive quantitative parameters by analyzing the curve. In this study, we aimed to derive useful parameters from EFVC via graphic analysis and tried to validate them in patients with COPD.

**Methods:**

Using Graphical Analysis 3.4 Vernier Software, we derived from the EFVC such parameters as area of obstruction (Ao), area of triangle (AT), area of rectangle (AR) and ratio of volume at 75 and 25 % peak expiratory flow (PEF) (0.25/0.75 V). For validation, we reviewed clinical and spirometric data of 61 COPD patients from Seoul National University Airway Registry (SNUAR) and Korean obstructive Lung Disease (KOLD) cohorts.

**Results:**

Of all parameters, only RV/TLC significantly correlated with scores from St. George’s Respiratory Questionnaire (SGRQ) (r = 0.447, *p* = 0.037). Six-minute walking distance (6MWD) highly correlated with Ao/AR (r = −0.618, *p* = 0.005) and Ao/PEF (r = −0.581, *p* = 0.009) whereas neither FEV_1_ nor FEV_1_/FVC had significant correlation with 6MWD.

**Conclusions:**

Ao/AR and Ao/PEF are promising parameters which correlate well with the exercising capacity of COPD patients.

**Electronic supplementary material:**

The online version of this article (doi:10.1186/s12890-016-0182-8) contains supplementary material, which is available to authorized users.

## Background

Chronic obstructive lung disease (COPD) is defined as having airflow limitation, measured as forced expiratory volume in one second (FEV_1_) divided by forced vital capacity (FVC). These conventional spirometric parameters, such as FEV_1_ or FVC, are currently accepted standards in grading the severity of airflow limitation; when FEV_1_/FVC is less than 0.70, diagnosis of COPD is made.

However, these parameters cannot exactly determine the health status of patients with COPD. For example, FEV_1_ shows weak association with physical activity and subjective symptom scores such as St. George’s Respiratory Questionnaire (SGRQ) [1–3]; little correlation between FEV_1_ and hospital readmission in such patients is also reported [4]. Therefore, dyspnea scores and objectively measured exercise capacity should also be considered and taken into account.

Exercise capacity, which can be simply measured by 6-min walking distance (6MWD), is reflective of activities of daily living and functional status of patients with COPD but shows poor correlation with FEV_1_ or FEV_1_/FVC [5, 6].

While it is well-known that the concave shape of expiratory flow-volume curve (EFVC) is suggestive of the presence of underlying small airway obstruction, few attempts have been made to quantify the concave area and correlate it with clinical indices in patients with COPD [7–9]. In this study, we sought to derive new useful graphic parameters from a commonly used spirometric curve and performed correlation analysis with symptom severity and exercise capacity. We initially hypothesized that the concave area under the flow-volume curve might reflect symptom severity and health status in patients with COPD.

## Methods

### Study subjects and general methods

The following study was approved by the institutional review board of Seoul National University Bundang Hospital (B-1108/134-004 and B-0508-023-009). Informed written consent for participation in the study was obtained from all participants.

For validation of new graphic parameters, we analyzed the clinical and baseline pre-bronchodilator best spirometric data of 61 COPD patients from the Seoul National University Airway Registry (SNUAR) and the Korean Obstructive Lung Disease (KOLD) cohort [10]. 40 patients from SNUAR and 21 patients from KOLD cohort were included in the analysis. The SNUAR cohort included stable COPD patients who were prospectively recruited from the outpatient pulmonary clinic of Seoul National University Bundang Hospital. The diagnosis of COPD was made according to the Global Initiative for Chronic Obstructive Lung Disease (GOLD) 2003 criteria. The KOLD cohort consisted of patients with stable COPD, who were prospectively recruited from the pulmonary clinics of 11 hospitals in Korea from June 2005 to September 2009.

Spirometry was performed using a Vmax 22 instrument (Sensor-Medics; Yorba Linda, CA, USA). Lung volumes were measured by body plethysmography (V6200; SensorMedics). Diffusing capacity for carbon monoxide (DLco) was measured by the single-breath method using a Vmax229D (Sensor-Medics). All pulmonary function tests were performed as recommended by the American Thoracic Society and European Respiratory Society.

### Graphic analysis methods

**Fig. 1:**
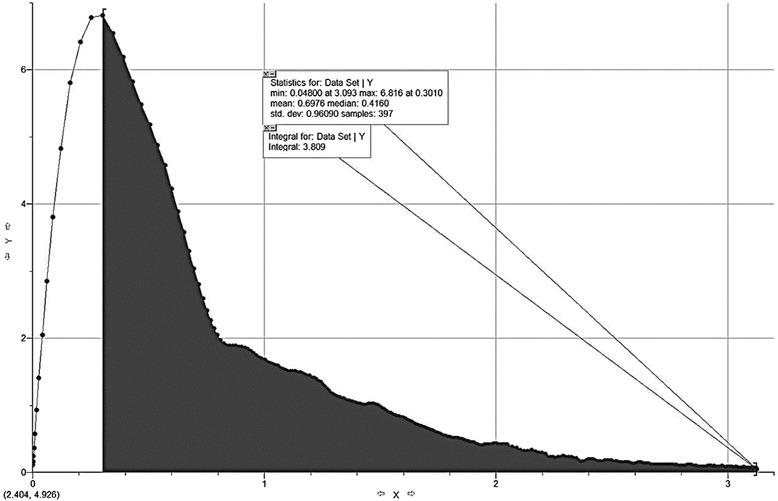
An example of maximal expiratory flow-volume curve and integration of the area under the curve

For graphic analysis of the EFVC, we used Graphical Analysis 3.4 Vernier Software program. Using the program, we first integrated the area under the curve. Figure 1 shows an example of pre-bronchodilator best EFVC and integration of the area under the curve using the software.

**Fig. 2:**
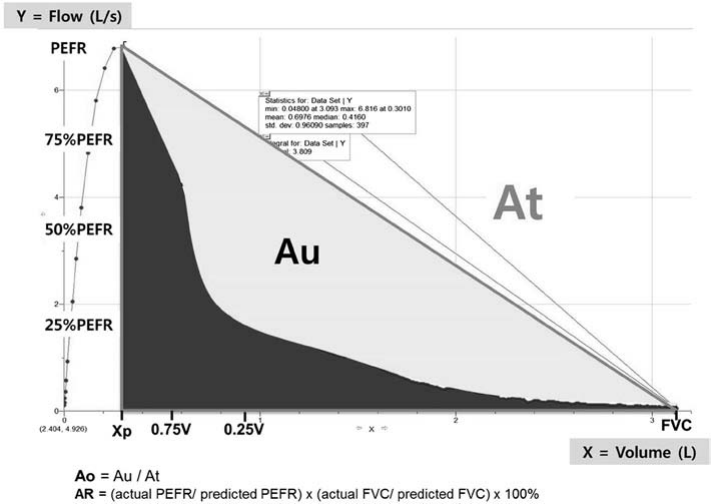
Graphic explanation of new parameters. Au was defined as the lighted area *below* the imaginary *diagonal line* and above the expiratory flow-volume curve. Area of obstruction (Ao) and area of triangle (At) were calculated as follows. Xp denotes the expirated lung volume at PEFR. The ratio of lung volumes at 75 and 25 % of PEFR (0.25/0.75 V) were also measured. Area of rectangle (AR), not shown here, was defined as follows. Ao = Au/At; At = PEFR × (FVC–Xp)/2; AR = (actual PEFR/predicted PEFR) × (actual FVC/predicted FVC) × 100 %

Figure 2 shows the lighted area below the imaginary diagonal line and above the EFVC; we defined this area as Au, which reflects concavity of the curve. Our new graphic parameter, Area of obstruction (Ao) and area of triangle (At) were calculated as follows.

$$ \mathrm{A}\mathrm{o}=\mathrm{A}\mathrm{u}/\mathrm{A}\mathrm{t} $$

$$ \mathrm{A}\mathrm{t}=\mathrm{PEFR}\times \left(\mathrm{F}\mathrm{V}\mathrm{C}\hbox{--} \mathrm{X}\mathrm{p}\right)/2 $$

As Ao approaches to 1, the concavity of EFVC becomes more severe. As Ao gets closer to 0, the EFVC becomes less concave. Xp denotes the lung volume at PEFR.

Because the baseline pulmonary function depends on the patients’ heights and other factors, we needed to define Ao in ratio rather than raw values. In order to calibrate and adjust for such individual differences, we divided Au by At. Using a raw value such as Au may result in wrong direction and inappropriate interpretation of result (see Additional file 1: Table S1).

Another parameter, area of rectangle (AR) were defined as follows; we used the reference equation derived from the Korea National Health and Nutrition Examinations Survey IV to calculate predicted FVC in Korean COPD patients [11, 12].

$$ \mathrm{A}\mathrm{R}=\left(\mathrm{actual}\kern0.5em \mathrm{PEFR}/\mathrm{predicted}\kern0.5em \mathrm{PEFR}\right)\times \left(\mathrm{actual}\kern0.5em \mathrm{F}\mathrm{V}\mathrm{C}/\mathrm{predicted}\kern0.5em \mathrm{F}\mathrm{V}\mathrm{C}\right)\times 100\% $$

The ratio of lung volumes at 75 and 25 % of PEFR (0.25/0.75 V) were also calculated.

### Statistical analysis

Statistical analysis was performed using the statistics software, PASW Statistics for Windows, Version 18.0, Chicago: SPSS Inc. The association between clinical and spirometric parameters was determined by using Pearson correlation analysis.

## Results

The demographic, clinical, and conventional spirometric characteristics are shown in Table 1. Data are presented as mean ± SD. The patients were mostly men (96.7 %) with mean age of 71 years old, 44 pack-years of smoking history and mean body mass index (BMI) of 23.4 kg/m^2^. Mean FEV_1_ was 65 % and FEV_1_/FVC 0.48.

**Table 1:** Clinical characteristics and conventional spirometric values of patients

	Mean (SD or %)	*N*
Age	71 (8)	61
Male	59 (96.7)	
BMI (kg/m^2^)	23.40 (3.40)	56
Smoking (pack-years)	44 (19)	59
FEV_1_/FVC	0.48 (0.12)	60
FEV_1_ (%)	65 (18)	60
DLco (%)	73 (20)	59
RV/TLC (%)	0.42 (0.11)	57
RV (%)	113 (43)	58
IC (L)	1.98 (0.56)	56
SGRQ (raw score)	1112 (571)	38
6MWD (m)	425 (126)	31
CAT	12.7 (7.7)	41

*Abbreviations*: *BMI* body mass index, *FEV*_*1*_ forced expiratory volume in one second, *FVC* forced vital capacity, *DLco* diffusing capacity for carbon monoxide, *RV* residual volume, *TLC* total lung capacity, *IC* inspiratory capacity, *SGRQ* St. George’s respiratory questionnaire, *6MWD* six-minute walking distance, *CAT* the chronic obstructive pulmonary disease assessment test

Among 61 subjects, the pre-bronchodilator EFVC data for three subjects were missing, so they were excluded in the correlation analysis. Table 2 displays the correlation result between clinical and known spirometric parameters after adjusting for age, BMI and smoking. Of all conventional parameters, only residual volume (RV) divided by total lung capacity (TLC) significantly correlated with SGRQ (r = 0.447, *p* = 0.037). Inspiratory capacity (IC), as percent predicted, showed positive correlation with 6MWD.

**Table 2:** Correlation analysis of traditional spirometric parameters and selected variables

	FEV_1_		FEV_1_/FVC		RV/TLC		PEF		IC	
	*r* ^a^	*P*-value	*r* ^a^	*P*-value	*r* ^a^	*P*-value	*r* ^a^	*P*-value	*r* ^a^	*P*-value
RV/TLC	−0.881 *	<0.001	−0.702 *	<0.001	–	–	−0.765 *	<0.001	−0.148	0.512
SGRQ	−0.355	0.105	−0.140	0.534	0.447 *	0.037	−0.306	0.202	−0.073	0.747
6MWD	0.201	0.371	0.042	0.853	−0.257	0.249	0.441	0.058	0.611 *	0.003
CAT	−0.226	0.313	−0.026	0.909	0.277	0.213	−0.233	0.337	−0.046	0.838

*Abbreviations*: *FEV*_*1*_ forced expiratory volume in one second, *FVC* forced vital capacity, *RV* residual volume, *TLC* total lung capacity, *IC* inspiratory capacity, *PEF* peak expiratory flow, *SGRQ* St. George’s respiratory questionnaire, *6MWD* 6-min walking distance, *CAT* the chronic obstructive pulmonary disease assessment test

^a^ *r*: Pearson’s correlation coefficient, adjusted for age, BMI and smoking

*P-value < 0.05

The results for new parameters derived from EFVC are given in Table 3. The new graphic parameters significantly correlated with RV/TLC were Ao, Ao/FVC, Ao/AR, and Ao/PEF.

**Table 3:** Correlation between new graphic parameters and selected variables

	Ao		AR		Ao/FVC		Ao/AR		Ao/PEF		PEF/(Xp-FVC)		0.25/ 0.75 V	
	*r* ^a^	*P*	*r* ^a^	*P*	*r* ^a^	*P*	*r* ^a^	*P*	*r* ^a^	*P*	*r* ^a^	*P*	*r* ^a^	*P*
RV/TLC	0.674*	0.001	−0.824*	<0.001	0.806*	<0.001	0.718*	0.001	0.780*	<0.001	−0.585*	0.004	−0.286	0.197
SGRQ	0.171	0.447	−0.432	0.065	0.422	0.072	0.388	0.101	0.408	0.083	−0.057	0.802	0.108	0.631
6MWD	−0.013	0.956	0.488*	0.034	−0.398	0.092	−0.618*	0.005	−0.581*	0.009	0.336	0.126	−0.097	0.667
CAT	0.161	0.475	−0.342	0.152	0.382	0.106	0.331	0.167	0.308	0.200	−0.035	0.876	−0.007	0.976

*Abbreviations*: *Ao* area of concavity, *AR* area of rectangle, *FVC* forced vital capacity, *PEF* peak expiratory flow, *Xp* the lung volume at peak expiratory flow, *0.25/0.75 V* ratio of volume at 75 and 25 % peak expiratory flow, *RV* residual volume, *TLC* total lung capacity, *SGRQ* St. George’s respiratory questionnaire, *6MWD* 6-min walking distance, *CAT* the chronic obstructive pulmonary disease assessment test

^a^ *r*: Pearson’s correlation coefficient, adjusted for age, BMI and smoking

*P-value < 0.05

Among clinico-physiological parameters, 6MWD highly correlated with Ao/AR (r = −0.618, *p* = 0.005) and Ao/PEF (r = −0.581, *p* = 0.009). Neither FEV_1_ nor FEV_1_/FVC had significant correlation with 6MWD.

## Discussion

We set out to determine whether new parameters could further reflect the clinical status of patients with COPD. In this study, we demonstrated that Ao/AR and Ao/PEF significantly negatively correlated with 6MWD. 6MWD is a simple, objective test of measuring functional capacity targeted at people with at least moderately severe impairment and can be used as a follow-up tool to show response after intervention. In addition, the new parameters, Ao/AR and Ao/PEF, highly correlated with RV/TLC, which is a measure of hyperinflation. On the other hand, conventional parameters, such as FEV_1_ or FEV_1_/FVC, did not show correlation with 6MWD in our study.

Most of previous studies sought to derive parameters reflecting concavity of the EFVC, but did not further investigate the correlation with clinical and functional indices. Mead et al. first developed the slop ratio as an index of the curvature of maximal EFVC, but it was regarded impractical to use [13]. Afterwards, studies conducted in asthma patients revealed that the concave shape of the curve became less bowed after the steroid treatment [14]; the relationship between the severity of wheezing and the concavity of EFVC was also suggested [15, 16]. In order to quantify the concavity, Vermaak et al. attempted to measure the area under the curve (Aex) as an imaginary triangle with FVC as the base, PEF the perpendicular axis, and straight descending portion of maximal EFVC as the hypotenuse, that seemed to be the most sensitive index to assess the degree of bronchodilation in patients with COPD [17, 18].

More recently, Nozoe et al. studied spontaneous expiratory (SEFV) curve of 34 stable COPD patients at resting state and found that the area under the curve divided by the surrounding rectangular area, the rectangular area ratio (RAR), is indicative of concavity of SEFV curve; FEV_1_ was the most powerful predictor of concavity of the curve [19]. Ma et al. also used the same parameter, RAR, in analysis of SEFV curve during exercise [20]. While RAR is an excellent novel parameter to measure concavity of the EFVC, these studies did not provide the data showing the relationship of RAR with symptom severity and exercise capacity. Another interesting pilot study by Williams et al. investigated tidal flow-time and flow-volume centroids of spirograms, which showed shorter time to reach PEF and more left-shifted centroid pattern in patients with COPD [21]. Assessing SEFV curve as in this recent study seems promising and is more applicable to elderly patients with comorbidities who cannot perform the maximal EFVC. Nevertheless, utilization of SEFV curve is not widely accepted yet in practice as much as the maximal EFVC. Future studies are warranted in examining SEFV curve to check the relationship between new parameters and functional indices.

The authors acknowledge that the present study has several limitations. First, the size of study sample was too small to fully validate our new parameters. Second, we analyzed the pre-bronchodilator best expiratory curves rather than the post-bronchodilator curves, which could have affected the curvature. Third, most patients were in moderate to severe stage of COPD, GOLD II-III, and we did not include the patients with mild COPD (FEV_1_ ≥ 80 %).

## Conclusion

In this study, we found that Ao/AR and Ao/PEF are potentially useful parameters which correlate well with exercising capacity in patients with COPD. Yet, further studies are warranted to validate the new parameters in a sample of sufficient size and also in SEFV curves.

## Additional file

Additional file 1: Table S1 Correlation analysis between Au and selected variables. Pearson correlation coefficients are shown in the left column with *p*-values in the right column. (DOC 30 kb)

## Abbreviations

COPD: chronic obstructive pulmonary disease
SNUAR: Seoul National University Airway Registry
KOLD: Korean Obstructive Lung Disease
EFVC: expiratory flow-volume curve
Au: area under the curve
Ao: area of concavity
AT: area of triangle
AR: area of rectangle
PEF: peak expiratory flow
FEV_1_: forced expiratory volume in one second
FVC: forced vital capacity
RV: residual volume
TLC: total lung capacity
Aex: the area under the curve
6MWD: 6-min walking distance
SGRQ: St. George’s Respiratory Questionnaire
BMI: body mass index
DLco: diffusing capacity for carbon monoxide
GOLD: Global Initiative for Chronic Obstructive Lung Disease
