# Erratum: Graphic analysis of flow-volume curves: a pilot study

**DOI:** 10.1186/s12890-016-0277-2

**Published:** 2016-08-08

**Authors:** Jungsil Lee, Choon-Taek Lee, Jae Ho Lee, Young-Jae Cho, Jong Sun Park, Yeon-Mok Oh, Sang-Do Lee, Ho Il Yoon, KOLD Study Group

**Affiliations:** 1 Department of Internal Medicine, Seoul National University College of Medicine, Division of Pulmonary and Critical Care Medicine, Seoul National University Bundang Hospital, 166, Gumi-ro, Bundang-gu, 463-707 Seongnam-si, Gyeonggi-do, Republic of Korea; 2 Department of Pulmonary and Critical Care Medicine and Clinical Research Center for Chronic Obstructive Airway Diseases, Asan Medical Center, University of Ulsan College of Medicine, Seoul, Republic of Korea; 3 Department of Pulmonary and Critical Care Medicine and Clinical Research Center for Chronic Obstructive Airway Diseases, Asan Medical Center, University of Ulsan College of Medicine, Seoul, Republic of Korea

## Erratum

The original version of this article [1] was unfortunately missing funding information within the acknowledgements section.

The work was Supported by grant no 11-2008-038 from the SNUBH Research Fund.
