# Supplementary material for: Graphic analysis of flow-volume curves: a pilot study
Source: BMC Pulm Med. 2016 Jan 22;16:18. doi: 10.1186/s12890-016-0182-8 (PMC4724104; doi:10.1186/s12890-016-0182-8)
Supplement: Additional file 1: Table S1 — Correlation analysis between Au and selected variables. Pearson correlation coefficients are shown in the left column with p-values in the right column. (DOC 30 kb) [file 12890_2016_182_MOESM1_ESM.doc]

**Table S1.** Correlation analysis between Au and selected variables. Pearson correlation coefficients are shown in the left column with p-values in the right column.

|  | Au | |
| --- | --- | --- |
|  | *r* ^a^ | *P*-value |
| RV/TLC | -0.625 | 0.002 |
| SGRQ | -0.312 | 0.158 |
| 6MWD | 0.457 | 0.032 |
| CAT | -0.240 | 0.281 |

*Abbreviations*: *Au* area under the curve.

^a^*r*: Pearson’s correlation coefficient, adjusted for age, BMI and smoking.
